# Presentation and care of a family with Huntington disease in a resource-limited community

**DOI:** 10.1186/s40734-017-0050-6

**Published:** 2017-04-12

**Authors:** Jarmal Charles, Lindyann Lessey, Jennifer Rooney, Ingmar Prokop, Katherine Yearwood, Hazel Da Breo, Patrick Rooney, Ruth H. Walker, Andrew K. Sobering

**Affiliations:** 1 New York, USA; 2 Department of Neurology, James J. Peters Veterans Affairs Medical Center, Bronx, NY USA; 3 Department of Neurology, Mount Sinai School of Medicine, New York City, NY USA

**Keywords:** Huntington disease, Trinucleotide repeat disorder, Anticipation, Cultural competency, Resource-limited community

## Abstract

**Background:**

In high-income countries patients with Huntington disease (HD) typically present to healthcare providers after developing involuntary movements, or for pre-symptomatic genetic testing if at familial risk. A positive family history is a major guide when considering the decision to perform genetic testing for HD, both in affected and unaffected patients. Management of HD is focused upon control of symptoms, whether motor, cognitive, or psychiatric. There is no clear evidence to date of any disease-modifying agents. Referral of families and caregivers for psychological and social support, whether to HD-focused centers, or through virtual communities, is viewed as an important consequence of diagnosis. The experience of healthcare for such progressive neurodegenerative diseases in low- and middle-income nations is in stark contrast with the standard of care in high-income countries.

**Methods:**

An extended family with many members affected with an autosomal dominantly inherited movement disorder came to medical attention when one family member presented following a fall. Apart from one family member who was taking a benzodiazepine for involuntary movements, no other affected family members had sought medical attention. Members of this family live on several resource-limited Caribbean islands. Care of the chronically ill is often the responsibility of the family, and access to specialty care is difficult to obtain, or is unavailable. Computed tomography scan of one patient’s brain revealed severe caudate atrophy and moderate generalized cortical atrophy. Genetic diagnosis of HD was obtained.

**Results:**

Through family recollection and by direct observation we identified four generations of individuals affected with HD. Outreach programs and collaborations helped to provide medical imaging and genetic diagnosis. Additionally these efforts helped with patient and family support, education, and genetic counseling to many members of this family.

**Conclusions:**

Affected members of this family have limited healthcare access, and rely heavily on family support for care. Genetic and clinical diagnosis of these patients was impeded by lack of resources and lack of access to specialty care. Importantly, obtaining a definitive diagnosis has had a positive impact for this family by facilitating genetic counseling, education, community outreach, and dispelling myths regarding this hereditary disease and its progression.

**Electronic supplementary material:**

The online version of this article (doi:10.1186/s40734-017-0050-6) contains supplementary material, which is available to authorized users.

## Background

### Huntington disease

Huntington disease (HD) is a neurodegenerative disorder typically of adult onset, thus affected individuals typically have children before symptom manifestation. Chorea and psychiatric symptoms are typical, however, language and memory of past events may remain intact in many patients [1, 2]. In late stages patients typically require increased care as they shift from their hyperkinetic chorea state, to a rigid hypokinetic phenotype [3], leading to an increased risk for depression among caregivers due to the stress associated with care for HD patients [4].

HD is an autosomal dominant neurological disorder caused by an expanded tandem array (>35 repeats) of CAG codons within exon 1 of the *HTT* gene [5, 6]. Intermediate repeats (26–35 repeats) are termed premutations, which have the potential to expand into the pathogenic allele during gametogenesis [7, 8]. An inverse relationship exists between repeat number and onset age of symptoms. The CAG repeat length tends to expand as the trait is passed down through inheritance, accounting for the earlier onset of symptoms in successive generations, known as anticipation. Largest expansions occur through male meiosis [9]. Many reviews describing HD exist, see [10, 11] for recent summaries.

Presymptomatic genetic diagnosis may inform at-risk individuals of their future disease state [12, 13]. There is considerable debate regarding the impact of advance knowledge of a future disease such as HD, as a positive result leads to increased risk for depression and suicide [14]. When presymptomatic testing for HD first became available, it was incorrectly predicted that the majority of at-risk individuals would want to know if they had the pathogenic mutation [15]. Different studies place the actual range of test use by at-risk individuals to be in the range of 12 to 25% [16, 17]. Indeed, most individuals who are at-risk for HD opt not to pursue genetic testing [18].

### Care of the HD patient in a resource-limited community

Care of a patient with HD is challenging regardless of the location and ideally should incorporate support from both public and charitable agencies [19]. Caregivers of patients with ongoing chronic disease experience stress, increasing their risk for depression due to the constant demands associated with caring for their affected loved ones. This stress is often compounded in low socio-economic status (SES) communities where healthcare systems are underfunded and not well developed [20, 21].

In this paper, we describe an extended family with many members affected with HD. The difficulties involved with care of these patients is compounded by their low SES status; barriers to access specialty, and even non-specialty, medical care; and the geographical isolation created by living on a small island nation. Apart from the initial presentation of the proband at the general hospital, all evaluations were performed on home visits which were welcomed by the patients and families. To our knowledge, this is the first report of genetically-confirmed HD in the West Indies, although three families of various ethnic backgrounds in Trinidad with clinically-diagnosed HD were reported more than 50 years ago [22]. In the broadly defined Caribbean region, the only reported genetically diagnosed cases of HD have come out of Cuba [23], and there appears to be a significant number of HD cases currently being studied in Puerto Rico (Sylvette Ayala Torres, PhD, University of Puerto Rico, and Zoé Cruz-Gil, President, Fundación Huntington Puerto Rico, personal communication). Examples of other genetic movement disorders diagnosed in the West Indies include the Huntington disease-like disorders [24], and cases of spinocerebellar ataxia [25]. Many other autosomal dominant chorea disorders are known and described in this recent comprehensive review [26].

### Location

This study was approved by a local IRB which is registered with the United States National Institutes of Health: patients gave consent for medical interviews, neurologic examination, photographs, videotaping, genetic testing, and documentation of family history. To protect privacy, we will not identify the precise location of the family or the Caribbean affiliation of the authors of this manuscript. The International Monetary Fund World Economic Outlook describes this country as an emerging market with a developing economy, ranking it in the lowest 10th percentile for gross domestic product with individual income at approximately $US 8000 per capita [27]. Major sources of employment are found in tourism, fishing, agriculture, and construction. For most of the population, nutrition is good, due to ready availability of fruit, vegetables, livestock, the local fishing industry, and imported food.

Medical care is provided at a local hospital, community health centers and privately practicing physicians. The community health centers provide general health care such as medications, blood pressure testing, wound care and dressings. The centers treat medical emergencies, acute situations, and provide advice regarding preventive care for conditions such as diabetes and hypertension. Importantly, medical care for ongoing or chronic illnesses such as HD is either nonexistent or difficult to obtain. In the general hospital neurological cases are typically referred to internal medicine (generalist) or, the patient must fly off-island because the community does not have a full-time practicing neurologist. In many cases, costs of medical care are covered by the government. However, patients must pay for the majority of prescription drugs, all non-urgent testing, and imaging that is not part of hospital admission. English is the official language of these islands; however many people of the native population speak in local dialect. The evaluating physicians are not native to the island, but have spent up to 25 years practicing in the community (>75 years combined) and are fluent in the dialect. The affected patients described in this report, as well as a significant percentage of the population, live in small, wooden, single-story buildings. Electricity and running water are found in most households but might be considered more basic compared to US or UK communities; many roads are unpaved or in a state of poor repair.

## Methods and Results

### An overview of the family

**Fig. 1:**
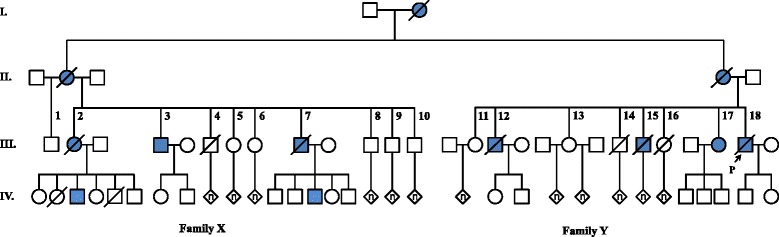
Four generation pedigree of family with many members affected with Huntington disease

During his initial medical evaluation and interview, the index case (III-18) described a number of other members of his extended family who also had a movement disorder. These other affected family members were sought out and three of those affected and many unaffected relatives agreed to be interviewed and examined. Based on their reported histories a detailed pedigree of the extended family was constructed (Fig. 1). All interviewed family members were asked about the possibility that they had Venezuelan heritage due to the high HD incidence in the Lake Maracaibo region [5] but we were unable to establish a connection. We note that individuals represented on this pedigree are now living on several different Caribbean islands, and also in the United States.

All affected individuals gave a similar history regarding their movement disorder in which control of their muscles seemed normal throughout childhood and early adulthood. All reported that their loss of muscle control developed at about 35 years of age. This was corroborated by unaffected family members as they recalled memories of their affected relations. During our interviews, the affected individuals appeared euthymic and did not exhibit frank psychiatric symptoms. They were conversant and we judged them to be using language appropriate for their level of education. Initially HD was presumed to be an unlikely diagnosis. This was based upon the apparent absence of cognitive or psychiatric impairment or lack of higher cerebral functions, and also that all family members agreed that the onset of chorea occurred at a similar age.

### Patient III-18

The index case was an approximately 50-year-old man who presented to the General Hospital. He had sustained a minor head injury and lacerations to the right side of his face and right arm as a result of falling while walking in the street. The patient had obvious chorea of his head and all four limbs which he said had caused him to fall. The patient insisted that falling in the street was unusual as he had learned how to live with his abnormal movements over the preceding ~20 years, and that despite his movement disorder he was usually able to maintain his balance.

The patient had been an accomplished athlete in his youth and hoped to pursue a career as a professional golfer. These aspirations were abandoned with the onset of his involuntary movements. At the time of presentation, the patient was gainfully employed as a gardener and enjoyed fishing. There was no evidence of a deterioration of performance in either work or hobbies, such as impaired ability to organize and perform specific tasks.

Cranial nerve examination revealed no abnormality apart from mild chorea of his face and his tongue. Speech was normal. His head and all four limbs displayed marked chorea. Tone was mildly reduced, and deep tendon reflexes were normal. Sensory examination was normal, and there was no evidence of cerebellar dysfunction.

The patient appeared to be oriented in time, place, and person. Based on his ability to interact and hold a conversation, his cognitive skills appeared consistent with his seventh grade education. This impression was supported by his physical presentation (personal hygiene, clothing), mood, language usage, and his ability to give an apparently appropriate detailed medical history, however, formal cognitive testing was not performed. Since the patient appeared stable, and a formal psychological testing battery was not available at this time, we did not pursue further cognitive investigations. The patient died within a year of this evaluation; the cause of death remains unknown.

### Patient III-17

This woman was examined initially at the age of approximately 48 years. She claimed to have developed involuntary movements approximately 12 years previously. Initially the family reported that there were no personality changes or psychiatric symptoms and her three children thought of her as a cornerstone of the family. She was regarded as a reliable, stalwart person, who could be counted on for counsel and wisdom.

On initial examination this patient had generalized chorea. Speech was fluent and not dysarthric. She was well-related, appeared to have generally high spirits, and was conversant. She was able to provide many details of the family tree, including her eight siblings and her eight cousins (the “X” family in the pedigree). Her memory appeared to be as good as, if not better than that of her unaffected relatives.

On follow-up two years later, her chorea had progressed and was so severe that it impaired her ability to perform ADLs. One year later it was clear upon casual conversation that her cognition had deteriorated. By the next year she was almost completely bedridden and markedly cachectic. She was alert and responsive but she spent most of her time lying on a mattress on the floor. She had moderately dysarthric speech, and was able to count from 1 to 10 forwards and backwards. She reported that three of her children were affected with the same movement disorder that she had, which was not accurate. She could perform simple tasks, such as finger-to-nose testing, accurately, but tended to perseverate. Power was intact in all limbs, and tone was normal. Deep tendon reflexes were increased throughout. Toes were downgoing. It was not possible to examine her eye movements due to neck chorea. She was able to push herself up to sit and stand alone, but with markedly impaired balance and required assistance for almost all ADL. She was prescribed lorazepam 1 mg b.i.d. from her family physician, which somewhat reduced the involuntary movements. We are currently considering further pharmacological strategies to control the involuntary movements, however, this is challenging due to the lack of ongoing neurologist support and difficulty accessing medications.

### Patient III-7

This patient had been employed as a caddy at the local golf course. He was well known by many in the community where most described him as a respectable, punctual, and kind person who was never talkative. On initial evaluation when he was approximately 46-years-old, he had mild chorea affecting his upper limbs and face, he was coherent and able to have a relatively normal conversation with respect to his normal non-verbal personality. On follow-up, three years later he had become severely rigid and hypokinetic, and was completely dependent for all ADLs. Even though he was always known to be reticent, by one year later he had ceased all efforts to verbally communicate and appeared apathetic.

On examination he was lethargic but arousable and somewhat attentive. With repeated questioning he would respond with one-word answers. Speech was moderately dysarthric. He was able to name his caregiver (his daughter-in-law), although he could not state her relationship to him. He was able to obey some, but not all, simple commands. He was unable to volitionally open his eyes, but actively resisted opening of his eyes by the examiner, with a Bell’s phenomenon. Tone was increased throughout, and deep tendon reflexes were brisk, with downgoing toes. Both arms were held in flexion with dystonic extension of some fingers. He was unable to extend his arms to perform finger-to-nose testing. He was unable to sit without assistance. He was severely cachectic with marked difficulty with eating and swallowing. A video showing this examination may be accessed via Additional file 1: Movie S1. Shortly after this last evaluation he was brought by his family to hospital for progressive deterioration, and subsequently died, although the cause was not determined.



**Additional file 1: Movie S1.** Video showing patient evaluation. This video shows subject III-7 in his home. (The low light conditions, even with light from the camera, reflect the absence of indoor lighting.) The patient is cachectic, with marked, generalized bradykinesia and rigidity. There is dystonic posturing of the right arm. He responds with one-word answers, and has difficulty performing simple commands. (MP4 11163 kb)


**Fig. 2:**
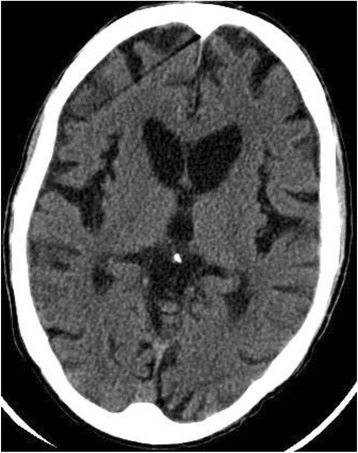
Brain CT scan of Patient III-7 showing generalized cortical atrophy and atrophy of the head of the caudate nucleus

Brain CT scan performed approximately six months prior to death revealed marked atrophy of the caudate nucleus and putamen (Fig. 2). A Mini Mental State Examination (MMSE) was performed, modified by the examiner to fit the cultural norms of the patient [28]. The patient was unable to proceed past the third question, and of the seven separate parts of those first three questions, he only scored one correct. Concerned that the patient might not feel at ease with the formal nature of the MMSE questions, the examiner attempted to engage him in conversation around his personal life and experiences. These impromptu, personalized and culture specific questions were directed towards determining the same five levels as the MMSE; orientation, registration, attention, recall and language. Once again, the patient’s responses suggested a poor level of cognitive functioning. During evaluation of this patient, we learned from another family member that when the patient first began showing movement disorder symptoms he would arrive for work at 4:00 a.m. rather than 6:30 a.m. This reported behavior is consistent with cognitive decline, specifically executive dysfunction affecting planning and decision-making [29].

Based on the family history, the phenotype of the affected family members, the CT scan, and the observed cognitive decline, we sought genetic testing to diagnose the disorder. Prior to offering the genetic test we counseled several members of the family about HD, including a discussion of possible outcomes and Mendelian genetic risk. We obtained genetic testing for HD (Neurodiagnostics Lab at Harvard Medical School) which revealed a pathogenically expanded trinucleotide repeat expansion of 47 repeats in one of the *HTT* alleles, and 17 repeats in the other.

## Discussion

The Caribbean island where this family was identified is small and resource-limited. Its economy is in a state of development and is based mainly on tourism. Because of financial limitations, medical service on the island is primarily focused on acute medical care, and attention to patients with chronic conditions lags behind that provided by most Western nations. While the island is home to physicians trained in reputable institutions world-wide, the opportunity for specialization is limited due to the small economy and population.

Without exception, all members of this extended family who we spoke to understood that there was an illness that tended to affect them. However, as far as we are aware, only one patient had ever sought medical attention for treatment and no family member had access to diagnostic services. Many of the adult children of affected subjects expected to inevitably develop the illness as they entered their mid-30s. We attempted to correct this misconception, and to educate members of this family about the Mendelian risk associated with autosomal dominant inheritance. This included the implications of delayed age of onset and how recurrence risk might be estimated in the grandchild of an affected family member.

Over several generations, reports from family members consistently suggested that age of symptom onset was occurring when the affected person was in their mid-30s. This stability might be attributed to the preponderance of maternal inheritance of the preceding generations. In the current generation we have now identified an affected individual in his mid-twenties who received the HD mutation paternally. This patient has not been formally evaluated by a neurologist. Variation of onset age due to paternal transmission of the HD trait is consistent with previous observations [9].

Affected subjects were taken care of in their homes by their family members. This mode of care is consistent with the level of medical care for many other chronic conditions in this environment. While patients with medically urgent conditions receive reasonable care, illnesses which are longstanding do not receive the same level of attention. Investigations such as imaging and laboratory tests have to be paid for by patients or their families. Genetic testing is not available locally. In this case, funds for the CT scan and genetic testing were raised by community outreach projects and fundraising events.

Initially, cognitive or psychiatric impairment was not apparent in the patients who were evaluated, and impairment was not noticed or reported by family members. For these reasons, HD was at first not in the differential diagnosis. Cognitive decline was not apparent to us until approximately three years passed from our initial contact with the affected individuals. Additionally, all of the patients terminated their education in their early teenage years, and their employment did not depend upon a high level of cognitive functioning. Because of the above, we had difficulty ascertaining cognitive decline. Additionally, subject III-17, who had marked chorea, was able to give extensive information about her family history going back three generations. Although it is difficult to validate her account, her recall of family lineage appeared to be excellent, and exceeded that of several other members of her family who were unaffected. The extensive family history provided by this patient led us to believe that her cognition was intact. This observation is consistent with reports that in some cases, patients with chorea tend to have less severe cognitive impairment [30] and that older memories tend to persist better then recent [31]. It is also possible that the index case and the two other affected members of the family who were encountered by the investigators were those members whose higher cognitive functions were least impaired, and who were able to maintain employment and other social interactions for a prolonged period despite their movement disorder.

Cultural differences may have also played a role in the ability of the authors to diagnose mood and cognitive status of the patients. Although some were raised in the West Indies or have spent considerable time on the island, the clinical investigators of this case are either of European descent or from developed nations. All the patients are of African descent and were raised in a small country with a developing economy. A dramatic disparity of SES exists between the investigators and these patients; these dynamics foster changes in the doctor-patient relationship to which both clinicians and patients are accustomed [32]. Traditional values are also important when considering neurological disorders involving involuntary movements and cognition changes. Examples can be found where HD patients and families on the border of Mexico and Texas had initially sought treatment from traditional faith healers because of perceived possession [33]. Most members of this West Indian family hold strong Christian religious values, and it is possible that without a medical reason to explain the condition, there was an unconscious withholding of information pointing to cognitive issues.

A hallmark of HD is onset of mood changes, psychosis, and suicidal thoughts prior to the presentation of the movement disorder. Due to local social conventions, it is possible that these patients and their families would not recognize, or would minimize, reports of mood changes, depression, or suicidal thoughts. Although directly asked about suicidal thoughts and depression, answers might not be forthcoming unless the question is framed within a precise social context, or possibly even by the correct person, and not by a clinician. It is also known that people with HD have limited insight into the extent and nature of both their movement disorder and their psychological impairment [34, 35]. An additional possibility is that both affected and unaffected family members perceived the illness within the context of the family lore, and ignored features which were inconsistent with this. The possibility exists that they may not have wanted to admit to depression for fear that the local stigma attached to mood disorders would render them ineligible for further help. Consistent with this were numerous reports of cognitive impairment in the affected individuals from various family members that were revealed only after the definitive diagnosis of HD was obtained. These observations included reports of sleep disturbances, paranoia, disorganization and asocial behavior that occurred at some point during the disease progression.

Following the confirmation of HD in patient III-7, several unaffected family members were informed of the diagnosis, and provided with information such as genetic features and age of onset. The response was uniformly positive, and it transpired that some had heard of HD, and this diagnosis had been discussed, although never confirmed. Even in the absence of further testing, unaffected children of affected subjects were reassured to learn that their chances of inheritance were 50:50, and that they couldn’t be affected “just a little bit”. Children of unaffected family members were reassured to learn that they would not develop the illness if their parent had not developed symptoms during a normal life span.

We were also able to discuss a misperception among members of the family regarding inheritance. At issue is the incorrect belief that a parent who is symptomatic during procreation or pregnancy might “cause” the trait to be passed on to her offspring. Since obtaining a definitive diagnosis, we have worked towards dispelling this myth, and to instead provide information regarding inheritance and progression of the disorder. Other at-risk individuals spoke of the disorder as being a “curse” that affects some members of the family. The idea of a curse is different from, but has parallels with how some cancer patients understand their disease [36]. Since many family members have internet access we were also able to direct family members to the appropriate on-line resources relevant to HD.

Until disease-modifying interventions for HD are identified, it could be argued that there is little benefit to confirming the diagnosis in subjects who are already affected in this population or in at-risk family members. Even when genetic diagnosis is available, many at-risk subjects choose not to learn of their carrier status. However, several family members studied here have expressed interest in learning of their carrier status and we are involved in discussions regarding the implications of presymptomatic diagnosis. This highlights the importance of cultural competency when discussing HD with at-risk individuals. The best way to discuss disorders such as HD with patients and their families in the West Indian region is currently an open question. In fact, there may not be a best practice for this region, because each island in the Caribbean archipelago is unique. In one case, we were asked about the possibility of presymptomatically providing the HD genetic test to a healthy 22-year-old woman who is at 50% risk. While we were considering how to provide the appropriate counseling services that should be associated with this test, the patient decided to opt out citing religious reasons. A secondary reason given was the idea that a consequence of testing might be manifestation of the disorder. Comparisons can be made with cancer: many studies reveal the importance of culture when discussing cancer such as the belief by some patients that talking about the disease will cause the disease [37, 38], and how family members and the community treat affected patients differently [39]. Currently no studies similar to these have been carried out in the Caribbean region with respect to Huntington disease.

## Conclusions

From our dealings with this family we believe that the patients and their at-risk relations appeared to benefit from the information we were able to provide regarding mode of inheritance, genetic aspects of pathology, and the understanding that there are other people in the world with the same disorder. We believe that for this family this is timely information, since there are approximately 20 at-risk individuals who we know of, and probably more, coming of age where symptoms might manifest. The value of proper genetic investigation in this family is clear despite the outcome being less sanguine than the initial medical assessments suggested, and supports pursuing genetic diagnoses even in this resource-limited environment.

## Abbreviations

ADL: Activities of daily living
HD: Huntington disease
*HTT*: Gene name abbreviation for huntingtin
MMSE: Mini mental state examination
SES: Socio economic status

## References

[1] Walker FO (2007). Huntington’s disease. Lancet.

[2] Tabrizi SJ, Scahill RI, Durr A, Roos RAC, Leavitt BR, Jones R (2011). Biological and clinical changes in premanifest and early stage Huntington’s disease in the TRACK-HD study: the 12-month longitudinal analysis. Lancet Neurol.

[3] Sturrock A, Leavitt BR (2010). The clinical and genetic features of Huntington disease. J Geriatr Psychiatry Neurol.

[4] Aubeeluck AV, Buchanan H, Stupple EJ (2012). ‘All the burden on all the carers’: exploring quality of life with family caregivers of Huntington’s disease patients. Qual Life Res.

[5] The Huntington’s Disease Collaborative Research Group (1993). A novel gene containing a trinucleotide repeat that is expanded and unstable on Huntington’s disease chromosomes. Cell.

[6] Brinkman RR, Mezei MM, Theilmann J, Almqvist E, Hayden MR (1997). The likelihood of being affected with Huntington disease by a particular age, for a specific CAG size. Am J Hum Genet.

[7] Yoon SR, Dubeau L, de Young M, Wexler NS, Arnheim N (2003). Huntington disease expansion mutations in humans can occur before meiosis is completed. Proc Natl Acad Sci U S A.

[8] Pearson CE (2003). Slipping while sleeping? Trinucleotide repeat expansions in germ cells. Trends Mol Med.

[9] Cleary JD, Pearson CE (2003). The contribution of cis-elements to disease-associated repeat instability: clinical and experimental evidence. Cytogenet Genome Res.

[10] Ross CA, Aylward EH, Wild EJ, Langbehn DR, Long JD, Warner JH (2014). Huntington disease: natural history, biomarkers and prospects for therapeutics. Nat Rev Neurol.

[11] Nopoulos PC (2016). Huntington disease: a single-gene degenerative disorder of the striatum. Dialogues Clin Neurosci.

[12] Williams JK, Erwin C, Juhl A, Mills J, Brossman B, Paulsen JS (2010). Personal factors associated with reported benefits of Huntington disease family history or genetic testing. Genet Test Mol Biomarkers.

[13] Tibben A (2007). Predictive testing for Huntington’s disease. Brain Res Bull.

[14] Wahlin TBR (2007). To know or not to know: a review of behaviour and suicidal ideation in preclinical Huntington’s disease. Patient Educ Couns.

[15] Hayden MR (2000). Predictive testing for Huntington’s disease: the calm after the storm. Lancet.

[16] Wedderburn S, Panegyres PK, Andrew S, Goldblatt J, Liebeck T, McGrath F (2013). Predictive gene testing for Huntington disease and other neurodegenerative disorders. Intern Med J.

[17] Morrison PJ, Harding-Lester S, Bradley A (2011). Uptake of Huntington disease predictive testing in a complete population. Clin Genet.

[18] Taylor SD (2004). Predictive genetic test decisions for Huntington’s disease: context, appraisal and new moral imperatives. Soc Sci Med.

[19] Banaszkiewicz K, Sitek EJ, Rudzińska M, Sołtan W, Sławek J, Szczudlik A (2012). Huntington’s disease from the patient, caregiver and physician’s perspectives: three sides of the same coin?. J Neural Transm.

[20] Schulz R, Sherwood PR (2008). Physical and mental health effects of family caregiving. Am J Nurs.

[21] Cohen S, Doyle WJ, Baum A (2006). Socioeconomic status is associated with stress hormones. Psychosom Med.

[22] Beauburn MH (1963). Huntington’s chorea in Trinidad. West Indian Med J.

[23] Vázquez-Mojena Y, Laguna-Salvia L, Laffita-Mesa JM, González-Zaldívar Y, Almaguer-Mederos LE, Rodríguez-Labrada R (2013). Genetic features of Huntington disease in Cuban population: implications for phenotype, epidemiology and predictive testing. J Neurol Sci.

[24] Stevanin G, Fujigasaki H, Lebre A, Camuzat A, Jeannequin C, Dode C (2003). Huntington’s disease-like phenotype due to trinucleotide repeat expansions in the TBP and JPH3 genes. Brain.

[25] Giunti P, Sabbadini G, Sweeney MG, Davis MB, Veneziano L, Mantuano E (1998). The role of the SCA2 trinucleotide repeat expansion in 89 autosomal dominant cerebellar ataxia families - frequency, clinical and genetic correlates. Brain.

[26] Hermann A, Walker RH (2015). Diagnosis and treatment of chorea syndromes. Curr Neurol Neurosci Rep.

[27] World Economic Outlook Database. http://www.imf.org/external/pubs/ft/weo/2014/02/weodata/index.aspx. Accessed 21 May 2016.

[28] Cockrell JR, Folstein MF. Mini-mental state examination. In: Copeland JRM, Abou-Saleh MT, Blazer DG, editors. Principles and practice of geriatric psychiatry. 2nd ed. John Wiley & Sons Ltd; 2002. p. 140–1.

[29] Rosenblatt A (2007). Neuropsychiatry of Huntington’s disease. Dialogues Clin Neurosci.

[30] Hart EP, Marinus J, Burgunder J, Bentivoglio AR, Craufurd D, Reilmann R (2013). Better global and cognitive functioning in choreatic versus hypokinetic-rigid Huntington’s disease. Mov Disord.

[31] Dumas E, van den Bogaard S, Middelkoop H, Roos R (2013). A review of cognition in Huntington’s disease. Front Biosci (Schol Ed).

[32] Willems S, De Maesschalck S, Deveugele M, Derese A, De Maeseneer J (2005). Socio-economic status of the patient and doctor-patient communication: does it make a difference?. Patient Educ Couns.

[33] Penaranda E, Garcia A, Montgomery L (2011). It wasn’t Witchcraft-it was Huntington disease!. J Am Board Fam Med.

[34] Hoth KF, Paulsen JS, Moser DJ, Tranel D, Clark LA, Bechara A (2007). Patients with Huntington’s disease have impaired awareness of cognitive, emotional, and functional abilities. J Clin Exp Neuropsychol.

[35] Sitek EJ, Soltan W, Wieczorek D, Schinwelski M, Robowski P, Reilmann R (2011). Self-awareness of motor dysfunction in patients with Huntington’s disease in comparison to Parkinson’s disease and cervical dystonia. J Int Neuropsychol Soc.

[36] Daher M (2012). Cultural beliefs and values in cancer patients. Ann Oncol.

[37] Karbani G, Lim JNW, Hewison J, Atkin K, Horgan K, Lansdown M (2011). Culture, attitude and knowledge about breast cancer and preventative measures: a qualitative study of south Asian breast cancer patients in the UK. Asian Pac J Cancer Prev.

[38] Thomas VN, Saleem T, Abraham R (2005). Barriers to effective uptake of cancer screening among black and minority ethnic groups. Int J Palliat Nurs.

[39] O’Callaghan C, Schofield P, Butow P, Nolte L, Price M, Tsintziras S (2016). “I might not have cancer if you didn’t mention it”: a qualitative study on information needed by culturally diverse cancer survivors. Support Care Cancer.

